# Instantaneous and Cumulative Knee Joint Loading in Cycling With and Without Medial Knee Osteoarthritis

**DOI:** 10.1111/sms.70259

**Published:** 2026-03-16

**Authors:** Jonas Ebbecke, Lasse Hansen, Josef Viellehner, William Brent Edwards, Nina Naeckel, Wolfgang Potthast

**Affiliations:** ^1^ Institute of Biomechanics and Orthopaedics German Sport University Cologne Cologne Germany; ^2^ Human Performance Laboratory, Faculty of Kinesiology University of Calgary Calgary Alberta Canada; ^3^ McCaig Institute for Bone and Joint Health, University of Calgary Calgary Alberta Canada; ^4^ Department of Biomedical Engineering University of Calgary Calgary Alberta Canada

**Keywords:** cumulative load modeling, cycling biomechanics, knee adduction moment, knee osteoarthritis, load modulation

## Abstract

Medial knee osteoarthritis (KOA) is characterized by altered joint loading that accelerates disease progression. Cycling is often recommended as a low‐impact exercise, yet the instantaneous and cumulative determinants of knee joint loading remain unclear. This study examined cyclists with and without KOA to characterize how power, cadence, and crank torque relate to knee load during cycling. Fifty‐six recreational cyclists (19 KOA, 19 age‐matched controls (CO), 18 younger controls (CY)) performed 3 cadences (60/80/100 rpm) at 3 power outputs (157/210/261W). Three‐dimensional kinematics and pedal reaction forces were used to compute knee moments via inverse dynamics. Instantaneous loading was quantified as peak knee adduction (KAM_peak_), flexion (KFM_peak_), and extension (KEM_peak_) moments. Cumulative exposure was assessed using fatigue‐weighted cumulative knee load (wCKL_KAM_, wCKL_KFM_, wCKL_KEM_) normalized to 1 h of cycling. Mixed‐effects linear models tested the effects of crank torque, power, and cadence on instantaneous and cumulative load. Crank torque best explained all instantaneous moments (all *p* < 0.001). KOA showed larger positive sensitivity to crank torque in KAM_peak_ than both control groups, but smaller torque‐related increases in KFM_peak_ (all *p* ≤ 0.01). For cumulative loading, power best explained wCKL_KAM_ and wCKL_KFM_, where opposing effects of instantaneous load and cycle count cancel the net effect of cadence. However, higher cadence at a given power was associated with greater wCKL_KEM_, particularly in KOA (*p* < 0.001). These results show that crank torque drives instantaneous knee loading, while power output and cadence govern cumulative exposure, highlighting the added value of cumulative metrics for characterizing differences in knee joint loading patterns between cyclists with and without KOA.

## 1 Introduction

Osteoarthritis is one of the most prevalent chronic joint diseases worldwide and is a source of considerable pain, disability, and loss of function. Knee osteoarthritis (KOA) is the most common form of osteoarthritis, with an estimated global prevalence of 365 million cases [1]. It is widely recognized as a whole‐joint disease that affects multiple tissues and leads to detectable changes in tissue structure, metabolism, and function [2]. In association with pathological tissue remodeling and inflammatory processes, osteoarthritis is strongly related to physical forces, causing damage to the articular cartilage and underlying subchondral bone [3].

Mechanical joint loading is therefore central to both the etiology and progression of KOA. Two components of the external knee moment are of particular relevance: the sagittal plane knee flexion/extension moment (KFM/KEM) and the frontal plane knee adduction moment (KAM) [4]. Sagittal plane moments largely dictate the overall level of joint loading [5], while KAM reflects the distribution of loads between the medial and lateral tibiofemoral compartments [6, 7]. Elevated knee joint moments, particularly higher KAM, during locomotion have consistently been associated with an accelerated progression of KOA [6, 7]. By contrast, sagittal‐plane moments are less consistently linked to structural worsening [4]. This suggests that individuals with KOA may experience greater increases in frontal‐plane loading with increasing external mechanical demand, whereas sagittal‐plane responses are expected to be more comparable across groups.

Despite the association between joint moments and KOA, regular exercise is critical for symptom relief, joint stability, and cartilage health [8, 9, 10, 11, 12, 13, 14]. Therefore, clinical guidelines from leading organizations strongly recommend exercise programs, including low‐impact activities, as a key treatment for KOA [15, 16, 17]. Cycling aligns well with these recommendations and has been shown to improve clinical outcomes in KOA [8, 11, 12, 18, 19, 20]. However, it is important to recognize that the knee is also the most common site for overuse injuries among cyclists [21, 22, 23]. This duality suggests that while certain cycling parameters can be therapeutic for KOA, others may predispose individuals to overuse injuries. To exploit the therapeutic potential of cycling while minimizing harmful load levels, it is essential to identify the mechanical determinants of knee joint loading across its planes of motion and relevant populations.

In real‐world scenarios, cyclists frequently encounter varying terrains and environmental conditions, resulting in a wide range of cadence and power output combinations. Their interaction directly determines crank torque, which influences pedal reaction forces and thereby knee joint loading. However, previous studies have only independently investigated the main effects of both parameters [5, 24, 25, 26]. For instance, Redfield and Hull [24] reported that knee joint moments are lowest near 105 rpm at a constant overall power output of 196 W. Fang et al. [5] systematically varied either power at a fixed cadence or cadence at a fixed power and found that higher power and lower cadence each increased knee joint moments across planes. Because these conditions were not combined in a full factorial design, the interaction between power and cadence has not yet been studied.

Building on this knowledge about healthy populations, a small number of studies have compared knee joint moments during cycling between individuals with KOA and age‐matched controls. Gardner et al. [27, 28] and Thompson et al. [29] reported no significant group differences in knee moments when participants cycled at a low‐intensity condition (60 rpm and 80 W). However, this single‐condition design does not allow conclusions to be drawn about how KOA and healthy cyclists differ in their sensitivity to loads when power and cadence are varied. Furthermore, in cycling as a repetitive movement, tissue loading results from the non‐linear interaction between load duration, magnitude, and number of cycles [30]. Reduced cadences at a constant power output can increase per‐cycle loading but simultaneously decrease the cycle count, which may offset the effects on cumulative exposure. To capture this, cumulative load models that weight the magnitude of joint moments relative to the number of loading cycles provide a more realistic representation of mechanical loading and potential tissue fatigue [30].

Taken together, the existing work has been restricted to per‐cycle loading metrics and largely limited to main‐effect analyses, leaving three important gaps: (a) how sagittal and frontal plane knee moments respond to different power × cadence combinations, (b) how these conditions influence not only instantaneous but also cumulative load exposure over time, and (c) whether individuals with KOA differ from healthy cyclists in their torque‐, power‐, and cadence‐related changes in knee joint loading.

The purpose of this study was therefore to characterize how instantaneous and cumulative knee joint loading during cycling vary with power output, cadence, and crank torque in individuals with and without medial tibiofemoral knee osteoarthritis. We specifically investigated how power output, cadence, and crank torque influence loading in both the sagittal and frontal planes. We hypothesized the following:

- Instantaneous knee joint loading during cycling is primarily determined by crank torque, exceeding the explanatory power of cadence or power alone.
- Weighted cumulative knee joint loading during cycling is primarily determined by power output due to opposing effects of cadence on per‐cycle load and cycle count.
- Compared to healthy controls, individuals with KOA are more sensitive to alterations of power output, cadence, and crank torque in the frontal plane, but not the sagittal plane.

## 2 Methods

### 2.1 Study Design

Fifty‐six participants were recruited and assigned to three groups: osteoarthritis (KOA), older controls (CO; age‐matched to KOA), and younger controls (CY; see Table 1). By including both an older, age‐matched control group and a younger control group, we were able to distinguish effects related to KOA from those related to age. Eligible participants were male or female recreational or competitive road or gravel cyclists (> 1 session/week, > 3000 km/year). Inclusion criteria for KOA were: age 40–65 years, a diagnosis of medial tibiofemoral KOA with a Kellgren–Lawrence [31] score of 2–4 (one or both sides), largely symptom‐free cycling, and no other musculoskeletal complaints. CO were aged 40–65 years, and CY 18–30 years. Cyclists with musculoskeletal complaints in the previous 3 months were excluded from both control groups.

**TABLE 1:** Participant demographics (mean ± SD).

	KOA	CO	CY
*N*	19	19	18
Female	4	3	6
Male	15	16	12
Age [years]	57.3 ± 6.7	54.1 ± 6.5	25.7 ± 6.9
Body mass [kg]	85.6 ± 17.4	76.9 ± 10.2	78.4 ± 10.1
Height [cm]	180.6 ± 9.7	180.7 ± 6.8	182.0 ± 10.0
BMI	26.1 ± 4.8	23.5 ± 2.6	23.6 ± 2.3
KL	2–4	—	—
Cycling experience [years]	21.4 ± 33.3	18.4 ± 11.6	7.4 ± 6.3
Cycling kilometers per year [km]	5915.8 ± 2823.8	6011.1 ± 1991.7	7200.0 ± 2901.5

Abbreviations: BMI, body mass index; CO, control group old; CY, control group young; KL, Kellgren–Lawrence Grade; KOA, Knee Osteoarthritis Group.

**FIGURE 1:**
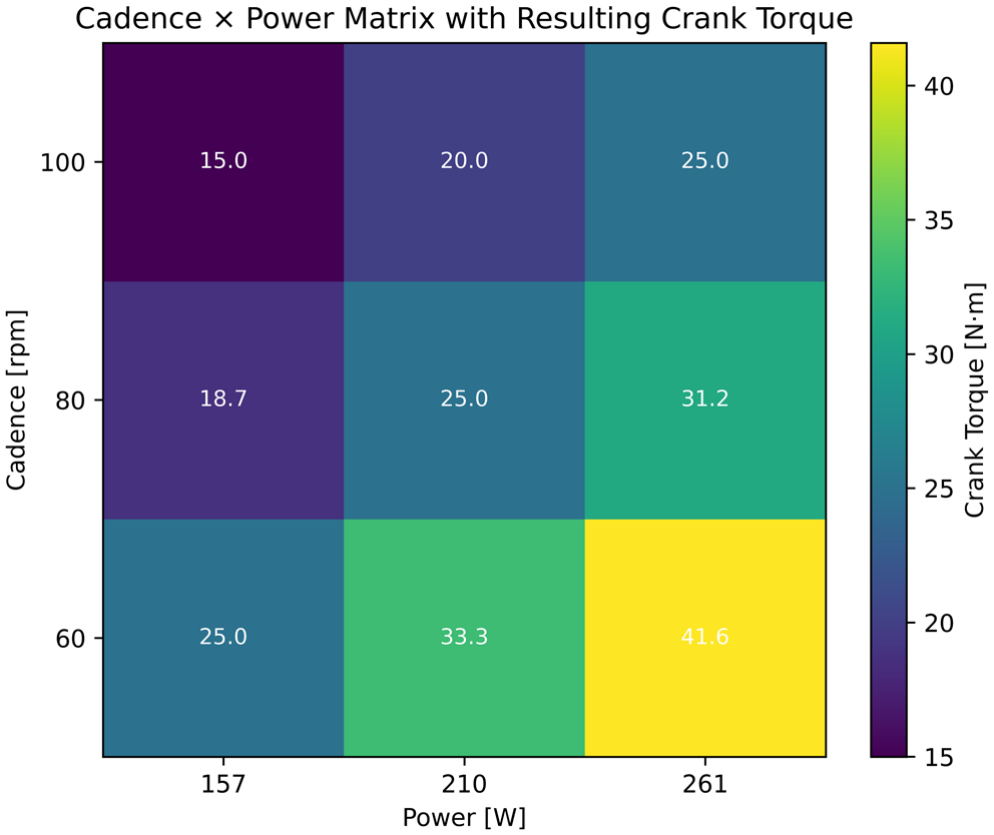
Cadence × power matrix and resulting crank torques. Each cell represents one of the nine cycling conditions combining three cadences (60, 80, 100 rpm) with three power outputs (157, 210, 261 W). Colors indicate the resulting crank torque, and the numeric values within each cell show the mean crank torque (N·m) for that condition.

The experimental design was a 3 (Group: KOA, CO, CY) × 3 (Cadence: 60, 80, 100 rpm) × 3 (Power: 157, 210, 261 W) factorial design. The resultant cadence × power matrix (Figure 1) was chosen to span a broad range of crank torques, including identical torque values along the diagonal, thereby allowing the individual effects of cadence, power, and crank torque on knee loading to be examined while holding the other factors constant. Independent variables were group (between‐subject factor), cadence, and power (within‐subject factor). The resulting crank torque was a derived mechanical determinant from cadence × power combinations. Dependent variables were the instantaneous and the fatigue‐weighted cumulative knee joint loading measures (defined in 2.4).

Before data collection, an a priori power analysis using G*Power (*F* tests—ANOVA: repeated measures, within–between interaction) was performed, based on a 3 (groups) × 9 (conditions) design. Assuming medium effect sizes (*f* = 0.25), *α* = 0.05, and desired power (1 − *β*) = 0.95, the required total sample size was *N* = 30. Because the final analyses were planned using mixed‐effects linear models, which typically require larger samples for stable estimation and the detection of interaction effects, we deliberately recruited beyond the minimum ANOVA‐based estimate. Our final sample size of *N* = 56 suggests that the study was a priori well powered to detect at least medium effect sizes. To verify that this a priori justification also holds for the mixed‐effects linear models used in the final analyses, we conducted Monte Carlo simulations based on the fitted models, which confirmed power (1 − *β*) > 0.8 for primary fixed effects and key group × determinant interactions.

This protocol was approved by the Ethics Committee of German Sport University Cologne (No. 211/2024) and was conducted in accordance with the Declaration of Helsinki. All participants provided written informed consent before participation.

### 2.2 Motion Capture and Inverse Dynamics

Lower body kinematics were measured using a 16‐camera motion capture system (Qualisys AB, Gothenburg, Sweden; 200 Hz) and the CAST lower body marker set [32]. Three‐dimensional pedal reaction forces were measured using custom‐made, instrumented cycling pedals at 2000 Hz. As described in detail by Ebbecke et al. [33], each pedal was instrumented with a three‐axis piezoelectric force sensor [Model 9251A, Kistler, Winterthur, Switzerland] and was dynamically calibrated and validated. Over the full calibrated range, the pedal shows a combined RMSE of 1.96 N (normalized RMSE 0.3%), with lower errors in cycling‐specific load ranges (normalized RMSE 0.04%). Bland–Altman analyses demonstrated biases close to zero and limits of agreement within ±4.04 N in all three loading directions.

Inverse kinematics and inverse dynamics were performed in OpenSim 4.5 [34], using the model proposed by Catelli et al. [35], which is optimized for tasks with substantial hip and knee flexion and therefore suitable for cycling. In its original form, the model comprises 37 degrees of freedom, including three rotational DOFs at the hip, one at the knee, and three at the foot. For the present study, the knee joint was implemented with three DOFs (flexion/extension, adduction/abduction, internal/external rotation) to allow calculation of three‐dimensional external knee moments.

The model was scaled non‐uniformly to each participant using OpenSim's Scale tool, based on a static calibration trial with anatomical landmark markers and virtual joint centers. Ankle and knee joint centers were defined according to ISB recommendations [36], and hip joint centers according to Harrington et al. [37]. Scaling quality was evaluated using the RMSE between experimental and model markers. Following OpenSim recommendations [38], solutions were accepted when the mean marker RMSE was below 10 mm, and the maximum error for bony landmark markers was below 20 mm. Scaling was therefore performed in a semi‐automated manner: an initial automatic solution was generated, and for participants whose marker residuals exceeded these thresholds, scaling parameters were manually adjusted until acceptable errors were achieved. Kinematic and kinetic data were low‐pass filtered with a zero‐lag, 4th‐order Butterworth filter (cut‐off 10 Hz) before inverse dynamics. The 10 Hz cut‐off for both data types was chosen based on exploratory frequency analysis, previous cycling‐related biomechanics literature [39, 40, 41], and to avoid filter‐induced inconsistencies when combining both signals in inverse dynamics [42].

### 2.3 Data Collection

All cycling trials were performed on an SRM ergometer (SRM GmbH, Jülich, Germany), equipped with road bicycle‐specific drop bars. Before data collection, the seat height was adjusted to match the recommendation of 25°–30° knee flexion angle at the bottom dead center during pedaling [43], with the knee joint center positioned vertically above the pedal at 90° crank angle. Retrospectively, the knee flexion angle at the bottom dead center was 28.5° ± 3.7°, and the knee joint center was 2.2 ± 24.9 mm in front of the pedal axis at 90° crank angle across all measured conditions. The height and reach of the handlebars were individually adjusted based on participant feedback, with participants confirming that the position was sportive but comfortable and similar to their usual riding setup. After a 10‐min warm‐up program with self‐selected power output and cadence, participants were asked to perform the nine cycling conditions in a randomized order. Prior to each recording, the target power and cadence were set in the SRMwin software, and the pedals' sensors were reset. The SRM ergometer automatically adjusts resistance to maintain the set power, which required participants to maintain the target cadence as accurately as possible while staying seated and having their hands at the hoods. Once the target cadence could be kept within a range of ±1 rpm, the recording was started for 30 s.

### 2.4 Knee Loading Parameters

All knee moments were expressed as external moments. Two different loading metrics were calculated for both the frontal plane (KAM) and the sagittal plane (KFM/KEM): (a) per‐cycle peak moments as a measure of instantaneous load magnitude, and (b) fatigue‐weighted cumulative moments as a measure of cumulative knee joint loads.

To quantify instantaneous loading, external knee moment–time curves were segmented into complete crank revolutions. The maximum adduction moment per cycle was defined as KAM_peak_. For the sagittal plane, the maximum flexion and extension moment was extracted as KFM_peak_ and KEM_peak_. Values were averaged across all valid revolutions for each participant and condition.

To quantify cumulative loading, we employed the mechanical fatigue approach described by Edwards [30], which models tissue damage as a non‐linear interaction between load magnitude, cycle number, and duration. Conceptually, material fatigue behavior can be described by Wöhler curves, in which the number of cycles to failure ($N_{f}$) is related to the applied stress (σ) via an inverse power‐law:

(1) $$N_{f}=A\cdot \sigma^{-b}$$

where A and b are material‐specific constants. If a material (tibiofemoral cartilage in the present case) is cyclically loaded with a task‐specific σ, and the number of cycles to failure is recorded, the material‐specific constants A and b can be determined by fitting Equation (1). Following Edwards [30], the exponent b can now be taken from the fitted function and used as a weighting factor when integrating joint loading over time, because b reflects how strongly the tolerable number of cycles decreases with increasing stress. The weighted integral for one cycle is then given by:

(2) $$\begin{array}{c} {\mathrm{wM}}_{\mathrm{int}}=\int_{t_{i}}^{t_{f}}M{\left(t\right)}^{b}dt \end{array}$$

where $M\left(t\right)$ denotes either the adduction, flexion, or extension moment, and $t_{i}$ and $t_{f}$ represent the start and end times of the loading cycle, respectively. Thus, higher moment magnitudes contribute disproportionately more to ${\mathrm{wM}}_{\mathrm{int}}$ than lower moments, consistent with the non‐linear fatigue behavior implied by Equation (1). To obtain cumulative values normalized to 1 h of cycling, the weighted integral is subsequently scaled by the number of cycles (n) corresponding to the condition‐specific cadence:

(3) $$\begin{array}{c} \text{wCKL}=n{\left[\int_{t_{i}}^{t_{f}}M{\left(t\right)}^{b}dt\right]}^{1/b} \end{array}$$

Here, wCKL denotes the weighted cumulative knee joint loading. The factor n scales the exposure linearly with the number of cycles (i.e., total duration at a given cadence). This formulation follows a modified version of the equation proposed by Edwards [30] (after personal correspondence with the author) to ensure linear scaling with the number of cycles while retaining non‐linear sensitivity to load magnitude.

Weighted cumulative loads were calculated separately for frontal‐plane moments (wCKL_KAM_) and sagittal‐plane moments (wCKL_KFM_, wCKL_KEM_). Following Miller and Krupenevich [44], who fitted the Equation (1) to tibiofemoral cartilage fatigue data as described above [45], we adopted *b* = 12.9 as the primary exponent. Because b is known to vary with tissue type and testing conditions, we performed a sensitivity analysis across the range *b* = 12.9 ± 4, which confirmed that the main findings were robust to the choice of exponent. All results reported in this manuscript are therefore based on *b* = 12.9.

### 2.5 Statistics

Mixed‐effects linear models (MLMs) were used to analyze instantaneous peak knee joint moments KAM_peak_, KFM_peak_, KEM_peak_, and cumulative loading measures wCKL_KAM_, wCKL_KFM_, wCKL_KEM_. MLMs accounted for the repeated‐measures design (nine cadence × power conditions per subject) and inter‐individual variability by including subject‐specific random intercepts.

For instantaneous loading, separate MLMs were fit with crank torque, power, or cadence as predictors, each including group (KOA, CO, CY), the predictor, and their interaction (group × predictor), as well as sex and body mass as covariates. For cumulative loading, the same approach was used, testing torque, power, or cadence as predictors of wCKL outcomes.

Model performances were compared using fixed‐effect estimates, *p*‐values, and Akaike Information Criterion (AIC) to evaluate the relative explanatory power of torque, power, and cadence. Effect sizes are expressed as unstandardized slopes (*β*), indicating the expected change in the outcome variable (Nm for instantaneous loads; Nm·s/h for cumulative loads) per one‐unit increase in the predictor (Nm for crank torque, W for power, rpm for cadence), while holding all other factors constant. All statistical analyses were performed using the statsmodels ([46], version 0.13.5) and custom code in Python (version 3.9.18).

## 3 Results

### 3.1 Instantaneous Knee Joint Loading

**FIGURE 2:**
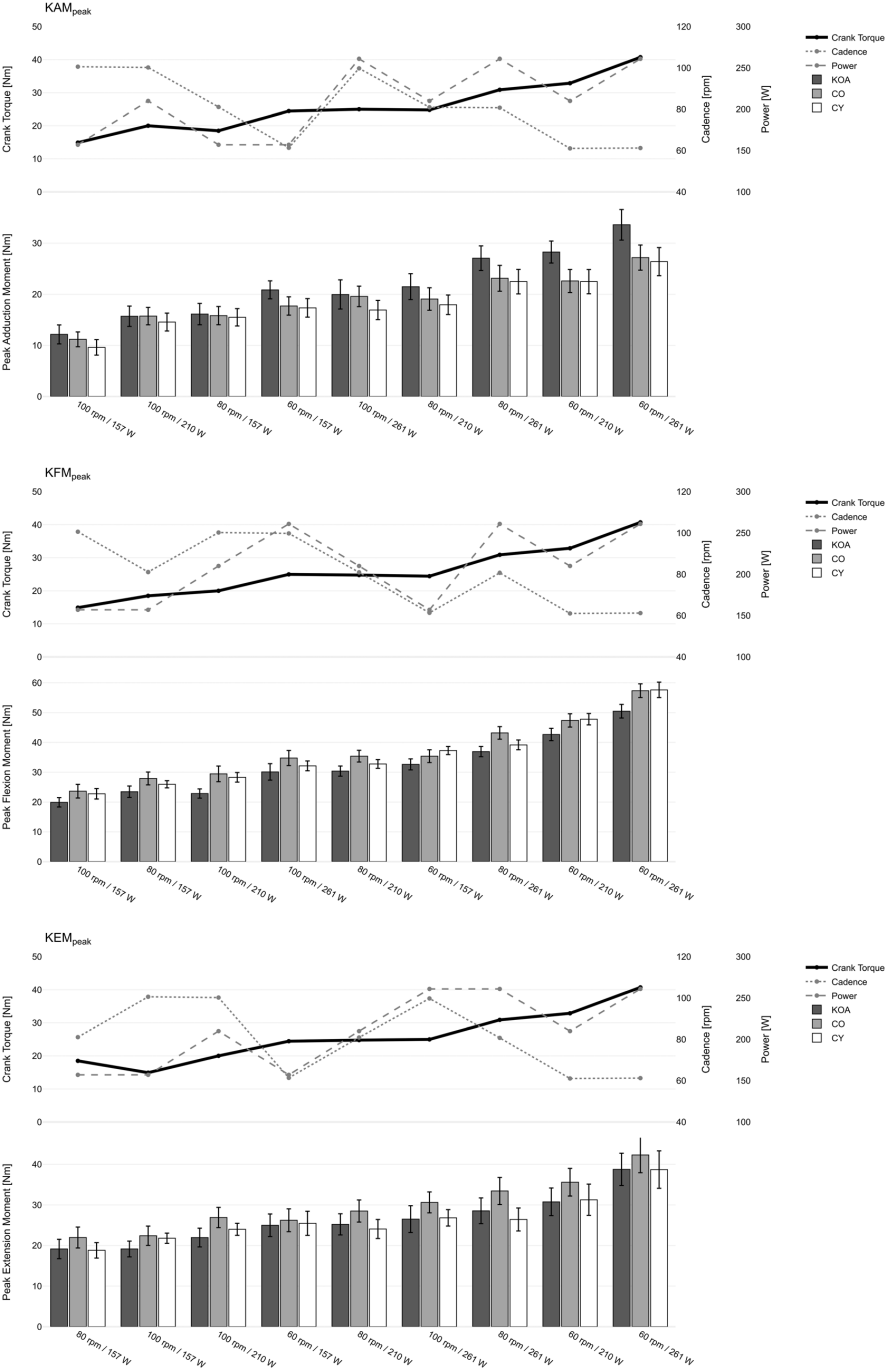
Instantaneous peak knee joint moments (KAM_peak_, KFM_peak_, KEM_peak_) across cycling conditions in participants with knee osteoarthritis (KOA), age‐matched controls (CO), and young controls (CY). Conditions are sorted by ascending mean peak moments across groups. Bars represent group means with standard errors. Line plots above each panel depict the corresponding cadence (rpm), power output (W), and resulting crank torque (Nm) for each condition. Thick line indicates best predictor for peak joint moments.

Across all three peak knee joint moments, models with crank torque as the predictor consistently provided the best fit compared to models using power or cadence, as indicated by lower AIC values (see Table 2; Figure 2).

**TABLE 2:** Mixed‐effects linear model results for instantaneous peak knee joint moments (KAM_peak_, KFM_peak_, KEM_peak_).

Outcome	Predictor	KOA		CO‐KOA		CY‐KOA		AIC
		*β*	*p*	Δ	*p*	Δ	*p*	
KAM_peak_	Torque	0.826	< 0.001*	−0.203	< 0.001*	−0.207	< 0.001*	2834.1^a^
	Cadence	−0.295	< 0.001*	0.104	0.003*	0.079	0.025*	3186.7
	Power	0.096	< 0.001*	−0.011	0.406	−0.018	0.178	3205.7
KEM_peak_	Torque	0.710	< 0.001*	0.046	0.638	−0.057	0.558	3438.1^a^
	Cadence	−0.214	< 0.001*	0.025	0.644	0.020	0.715	3607.4
	Power	0.092	< 0.001*	0.017	0.393	−0.011	0.584	3558.0
KFM_peak_	Torque	1.166	< 0.001*	0.144	0.041*	0.182	0.01*	3108.3^a^
	Cadence	−0.436	< 0.001*	−0.002	0.970	−0.073	0.194	3571.9
	Power	0.125	< 0.001*	0.026	0.288	0.012	0.628	3703.3

*Note:* Reported are KOA group slopes (*β*) with *p*‐values, group slope differences (Δ) relative to KOA with corresponding interaction *p*‐values, and model fit indices (AIC).

Abbreviations: AIC, Akaike Information Criterion; CO, control group old; CY, control group young; KAM_peak_, peak knee adduction moment; KAM_peak_, peak knee flexion moment; KEM_peak_, peak knee extension moment; KOA, Knee Osteoarthritis Group.

^a^ Best model fit.

* *p* < 0.05.

For KAM_peak_, the torque model showed a positive slope in KOA participants (*β* = 0.83 Nm/Nm, *p* < 0.001). Significant group × torque interactions indicated flatter slopes for CO (*β* = −0.20 Nm/Nm, *p* < 0.001) and CY (*β* = −0.21 Nm/Nm, *p* < 0.001) relative to KOA. Thus, increases in crank torque were associated with larger increases in KAM_peak_ in KOA than in both control groups. In comparison, the power and cadence models fit less well, although both predictors were significant (power: *β* = 0.096 Nm/W, *p* < 0.001; cadence: *β* = −0.29 Nm/rpm, *p* < 0.001).

For KEM_peak_, the torque model again showed the best fit (*β* = 0.71 Nm/Nm, *p* < 0.001). No significant group × torque interactions were observed. Models with power (*β* = 0.092 Nm/W, *p* < 0.001) or cadence (*β* = −0.21 Nm/rpm, *p* < 0.001) as predictors demonstrated higher AIC values.

For KFM_peak_, the torque model again showed the lowest AIC and a strong positive slope for KOA (*β* = 1.17 Nm/Nm, *p* < 0.001). Group × torque interactions were significant, with steeper slopes for both CO (*β* = 0.14 Nm/Nm, *p* = 0.041) and CY (*β* = 0.18 Nm/Nm, *p* = 0.010) relative to KOA, revealing greater sensitivity of KFM_peak_ to changes in crank torque in both control groups compared with KOA. Power (*β* = 0.13 Nm/W, *p* < 0.001) and cadence (*β* = −0.44 Nm/rpm, *p* < 0.001) were also significant predictors but again showed a poorer model fit.

### 3.2 Cumulative Knee Joint Loading

**FIGURE 3:**
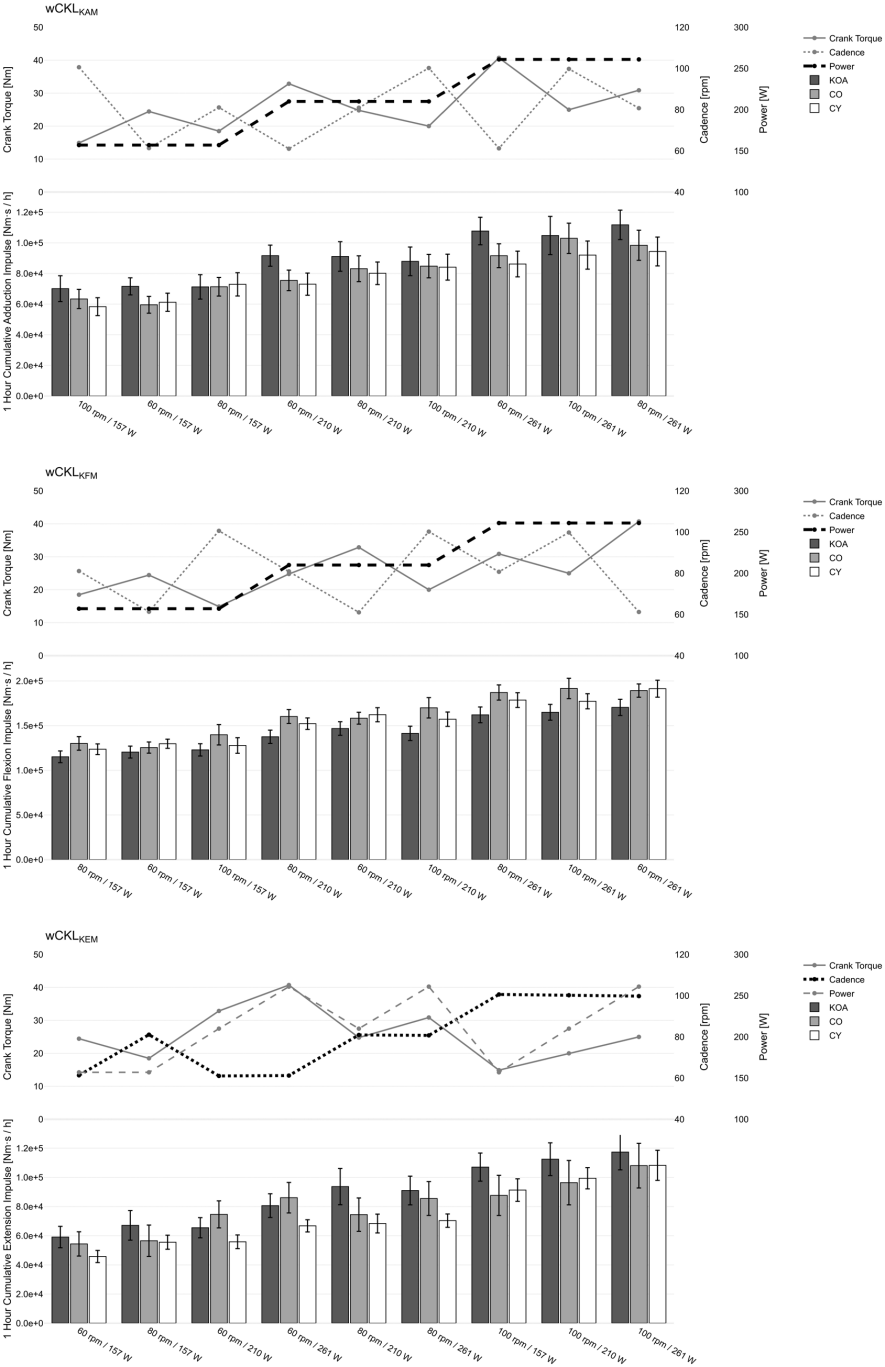
Weighted cumulative knee joint moments (wCKL_KAM_, wCKL_KFM_, wCKL_KEM_) across cycling conditions in participants with knee osteoarthritis (KOA), age‐matched controls (CO), and young controls (CY). Conditions are sorted by ascending mean wCKL across groups. Bars represent group means with standard errors. Line plots above each panel depict the corresponding cadence (rpm), power output (W), and resulting crank torque (Nm) for each condition. Thick line indicates best predictor for wCKL.

Model fit indices (AIC) indicated that power best explained wCKL_KAM_ and wCKL_KFM_, while cadence best explained wCKL_KEM_ (Table 3 and Figure 3).

**TABLE 3:** Mixed‐effects linear model results for weighted cumulative joint moments (wCKL_KAM_, wCKL_KEM_, wCKL_KFM_).

Outcome	Predictor	KOA		CO‐KOA		CY‐KOA		AIC
		*β*	*p*	*Δ*	*p*	*Δ*	*p*	
wCKL_KAM_	Torque	1409.6	< 0.001*	−401.8	0.131	−607.3	0.023*	11231.3
	Cadence	−58.6	0.552	235.5	0.087	171.7	0.215	11319.0
	Power	339.6	< 0.001*	−6.6	0.856	−72.1	0.051	11017.2^a^
wCKL_KEM_	Torque	−1062.4	< 0.001*	1031.4	0.012*	197.6	0.632	11630.8
	Cadence	1200.3	< 0.001*	−464.6	0.003*	−98.5	0.529	11447.0^a^
	Power	158.5	0.002*	79.8	0.259	15.3	0.831	11609.2
wCKL_KFM_	Torque	1854.5	< 0.001*	−22.9	0.948	551.2	0.120	11465.7
	Cadence	−90.9	0.523	370.4	0.062	−97.4	0.626	11626.7
	Power	421.2	< 0.001*	124.3	0.008*	112.4	0.017*	11215.6^a^

*Note:* Reported are KOA group slopes (*β*) with *p*‐values, group slope differences (*Δ*) relative to KOA with corresponding interaction *p*‐values, and model fit indices (AIC).

Abbreviations: AIC, Akaike Information Criterion; CO, control group old; CY, control group young; KOA, Knee Osteoarthritis Group; wCKL_KAM_, weighted cumulative knee adduction moment; wCKL_KEM_, weighted cumulative knee extension moment; wCKL_KFM_, weighted cumulative knee flexion moment.

^a^ Best model fit.

* *p* < 0.05.

For wCKL_KAM_, the power model showed the lowest AIC and a strong positive slope in KOA (*β* = 339.6 Nm·s/h per W, *p* < 0.001). Group × power interactions were not significant. Despite its weaker model fit, the torque model still showed a significant positive slope (*β* = 1409.6 Nm·s/h per Nm, *p* < 0.001), with a steeper slope for KOA compared to CY (*β* = −607.3 Nm·s/h, *p* = 0.023). The cadence model did not yield a significant predictor effect.

For wCKL_KEM_, the cadence model provided the best fit (*β* = 1200.3 Nm·s/h per rpm, *p* < 0.001). A significant group × cadence interaction indicated flatter slopes for CO compared to KOA (*β* = −464.6 Nm·s/h, *p* = 0.003). Thus, increases in cadence produced greater increases in cumulative extension loading in KOA than in age‐matched controls. Power and torque also reached significance (*p* < 0.001), but with higher AIC values.

For wCKL_KFM_, the power model again showed the best fit (*β* = 421.2 Nm·s/h per W, *p* < 0.001). Group × power interactions indicated steeper slopes for both CO (*β* = 124.3 Nm·s/h, *p* = 0.008) and CY (*β* = 112.4 Nm·s/h, *p* = 0.017) compared to KOA. Accordingly, cumulative flexion loading increased more with power in both control groups than in KOA. The torque was also significant but showed poorer model fit, while cadence was not significant.

## 4 Discussion

This study aimed to understand per‐cycle and mechanical fatigue‐weighted cumulative knee joint loading during sportive cycling with varying power and cadence conditions in individuals with and without KOA. For this purpose, three hypotheses were formulated: (a) crank torque is the primary determinant of instantaneous knee joint loading during cycling; (b) power output is the primary determinant of cumulative knee joint loading during cycling; (c) individuals with KOA are more sensitive to alterations of power output and cadence in the frontal plane, but not the sagittal.

### 4.1 Determinants of Instantaneous Knee Joint Loading

The peak knee moments observed in our study were comparable to those reported by Redfield and Hull [24], despite their use of a different musculoskeletal modeling approach. While most of our peak moments were generally higher than those reported by Fang et al. [5], the isolated effects of cadence and power showed consistent trends: higher cadence at a given power output led to lower peak knee joint moments, whereas higher power output at a given cadence led to higher peak knee joint moments across all planes. Direct comparison to Fang et al. is limited, as workloads were expressed in kilograms, making translation to mechanical power output difficult. While these earlier studies examined the main effects of cadence or power in isolation, our MLM approach built on this foundation by offering deeper insight into the determinants of instantaneous loading. Our MLM analyses confirm prior findings [5, 24, 25] by showing that cadence and power each significantly influence peak joint moments, as indicated by significant fixed‐effect slopes in all models. However, crank torque consistently provided superior explanatory power, as reflected by substantially lower AIC values across all peak moment outcomes. This confirms our hypothesis (a): Instantaneous knee joint loading during cycling is primarily determined by crank torque, exceeding the explanatory power of cadence or power output alone.

These findings align with biomechanical reasoning: power output is the product of cadence and crank torque, and thus power and cadence are physically interdependent. The isolated effects of cadence and power can therefore be misleading when modeled independently of each other. Crank torque, however, provides a more direct descriptor of the external mechanical demand at the crank. Under typical cycling coordination, where the ankle primarily serves force transmission and is stiffened through the plantar flexors [25, 47], this demand is transferred from the crank to the knee joint and scales proportionally with the external moments at the knee. Overall, the results indicate that while cadence‐ or power‐based models offer valuable insights into the effects on knee joint moments [5, 24, 25, 26], they potentially oversimplify the mechanics of per‐cycle knee joint loading. Instead, crank torque provides a more direct and robust determinant of instantaneous joint loading during cycling.

### 4.2 Determinants of Cumulative Knee Joint Loading

While cumulative load has been considered in other biomechanical contexts mainly during running [44, 48, 49, 50], to our knowledge, no study has applied a fatigue‐weighted cumulative load model to knee joint loading in cycling. Applying this novel approach in the present study, we found that power output was the most consistent determinant of cumulative joint loading across planes. For wCKL_KAM_ and wCKL_KFM_, power output was the strongest and most consistent predictor. In both cases, the power‐only model provided the best overall fit, and power slopes were statistically significant. Crank torque also showed a significant effect but explained less variance. In contrast, cadence did not significantly predict either outcome, and its models showed the poorest fit. This absence of a cadence effect can be explained by its counteracting influences on cumulative exposure: lower cadence at constant power output increases the load per cycle but simultaneously reduces the number of cycles performed, while higher cadence reduces the per‐cycle load but increases cycle count. Over time, these opposing effects cancel out, such that cadence does not substantially influence cumulative adduction or flexion moments. These findings support Hypothesis (b), emphasizing that cumulative loading is best captured by power, which incorporates both force magnitude and temporal exposure.

For wCKL_KEM_, however, a different pattern emerged. Here, cadence was the best predictor, with a significant positive slope. A likely explanation for this cadence effect lies in pedaling mechanics: at high cadences, many cyclists produced peak pedal reaction forces later in the crank cycle, such that vertical forces are still prominent after the bottom dead center [39], a pattern that was also qualitatively observed in the present data. This temporal shift in force application increases knee extension moments and, over time, amplifies cumulative extension loading.

Taken together, these results refine Hypothesis (b): while cumulative adduction and flexion moments are primarily determined by power output, cumulative extension loading is better explained by cadence. This highlights that cumulative exposure is not uniformly governed by a single parameter across planes, but instead depends on the interplay of power, torque, and cadence with plane‐specific load mechanics. Importantly, the divergence between instantaneous peak loading and cumulative loading underscores the added value of cumulative measures: whereas peaks quantify maximum per‐cycle loading, cumulative models integrate both magnitude and temporal repetition, and thus more realistically capture the total mechanical exposure experienced by knee tissues during cycling.

### 4.3 Group Differences in Instantaneous and Cumulative Loading

For instantaneous loading, crank torque revealed clear plane‐specific group differences. For KAM_peak_, the torque‐moment slope was highest in KOA, and significantly lower in both CO and CY. Across the tested torque range, each additional 10 Nm of crank torque increased KAM_peak_ by about 8.3 Nm in KOA but only by about 6.2 Nm in the control groups, i.e., KOA showed roughly 30% greater torque sensitivity of frontal‐plane loading. In contrast, for KFM_peak_, the torque‐moment slope was lower in KOA than in both control groups, which showed steeper slopes. For a 10 Nm increase in torque, KFM_peak_ rose by about 11.7 Nm in KOA compared with approximately 13–13.5 Nm in the control groups, indicating a modest but statistically significant greater torque sensitivity of sagittal‐plane flexion loading for both CO and CY. For KEM_peak_, torque slopes were similar across groups, suggesting comparable torque‐related changes in extension moments. Taken together, these findings suggest that KOA cyclists respond to increased crank torques with a relatively stronger shift toward medial knee compartment loading, whereas both control groups accommodate higher torques primarily by increasing sagittal‐plane flexion moments. This interpretation is consistent with prior studies by Thompson et al. [29], who reported that individuals with medial KOA exhibit altered frontal‐plane knee loading compared with healthy controls during cycling. They also extended these moment‐based findings to mediolateral tibiofemoral contact force patterns and demonstrated that group differences persist across different cycling conditions and reflect disease‐specific load redistribution strategies rather than simple scaling of external mechanical demand. Building on these findings, the largely similar instantaneous patterns observed in CO and CY in the present study suggest that the observed group differences are more likely related to the presence of KOA than to age alone, consistent with hypothesis (c) for instantaneous loading.

For cumulative loading, group differences were generally smaller and followed a slightly different plane‐specific pattern. For wCKL_KAM_, power was the primary determinant across all groups. KOA showed a strong positive power–wCKL_KAM_ relationship, and the corresponding slopes in CO and CY were very similar, indicating that cumulative frontal‐plane loading increased with power to a comparable extent in all groups. For wCKL_KFM_, however, the power–wCKL_KFM_ slope was significantly steeper in both control groups than in KOA. Compared with KOA, the increase in cumulative flexion loading per Watt was about 25%–30% higher in CO and CY. Thus, both control groups accumulated sagittal‐plane flexion loading more rapidly with increased power than KOA, which mirrors the instantaneous KFM_peak_ findings. For wCKL_KEM_, cadence was the best predictor, and here group differences were more pronounced. KOA showed a strong positive cadence–wCKL_KEM_ relationship, and the corresponding slope was significantly smaller in CO (approximately 40%) but not different from younger controls. This indicates that cumulative extension loading increased more with cadence in KOA and younger controls than in older controls. Possible reasons include differences in neuromuscular capacity, muscle strength, pedaling technique, or disease‐related movement strategies, such as altered inter‐joint load redistribution between hip, knee, and ankle. Gait studies during walking‐based tasks (e.g., level walking, ramp, and stair ascent/descent) have reported combinations of increased hip flexion moments and reduced knee flexion moments in people with medial KOA compared with controls, which have been interpreted as a proximal shift of sagittal‐plane loading toward the hip [51, 52, 53]. Such adaptations may reflect both compensatory and constrained neuromuscular strategies and are likely to be task dependent. In the context of cycling, it is therefore conceivable that some individuals with KOA may be limited in how much they can further adjust hip and ankle contributions when cadence increases, resulting in a greater proportion of the load being borne at the knee. However, hip and ankle joint moments were not analyzed in the present study, and the cross‐sectional design does not allow us to establish causal relationships between such inter‐joint strategies and the observed knee loading patterns.

### 4.4 Practical Implications

The distinct determinants of instantaneous and cumulative knee joint loading in cycling have direct clinical and applied implications. Increasing power output elevated cumulative sagittal‐plane moments (and therefore overall knee compression) and shifted frontal plane loading toward the medial compartment. Especially for individuals with KOA, this highlights that reducing overall power output is the most effective strategy to limit knee joint load accumulation, even though an instantaneous perspective might suggest that simply increasing cadence at a given power would be beneficial. For healthy controls, the same reasoning applies, where prolonged high‐power sessions lead to substantially high cumulative knee loading, and this accumulation is not meaningfully reduced by choosing higher cadences.

The cadence effect observed for extension moments underlies a different mechanism: a temporal shift in force application increases knee extension moments and, over time, amplifies cumulative extension loading. Practically, this suggests that the combination of high cadence and high power elevates both overall knee compression and medial load shift. Caution is therefore advised with this combination in individuals with tibiofemoral knee osteoarthritis, who are often considered to be sensitive to joint loading, although the available data do not allow the establishment of threshold values for excessive or harmful loading. At the same time, it also identifies a target for intervention: prior studies [54, 55, 56] demonstrated that pedal force profiles can be modified through biofeedback and neuromuscular training. Building on this, interventions such as cadence‐focused drills, feedback on pedal force orientation and timing, or biofeedback training could help cyclists optimize pedal force production for reduced knee joint moments, also at high cadences.

This leads to the conclusion that KOA patients may benefit from adopting moderate cadences and lower power outputs. Where higher cadences are desired, cyclists may focus on pedaling techniques that reduce late vertical force after bottom dead center and promote a more tangential and earlier‐applied pedal force profile. For healthy cyclists, analogous refinements in the timing and orientation of pedal forces at high cadences could reduce cumulative knee joint loading and may enhance pedaling efficiency. More broadly, our findings highlight that cumulative load models capture aspects of joint exposure that are not reflected in instantaneous peaks. Considering both approaches together may therefore provide a more comprehensive basis for tailoring exercise prescriptions for knee joint health.

### 4.5 Limitations

This study has several limitations. First, the experimental protocol relied on controlled ergometer conditions with prescribed cadence and power output, which may not fully reflect real‐world cycling, where cadence and power can fluctuate in response to terrain and rider input. Consequently, the relationships between cadence, power, and crank torque observed here should be interpreted as specific to controlled conditions. Second, participants were exclusively active cyclists, limiting generalizability to less active individuals with KOA. Although all groups cycled at identical absolute mechanical workloads, relative physiological effort (e.g., relative to individual aerobic capacity) was not assessed and may have differed between participants; however, this does not affect the external mechanical demands that determine knee joint loading. Third, although wCKL was developed for repetitive stress and strain, it was applied here to knee joint moments. While these are established proxies for compressive forces and medial load distribution, this approach does not directly estimate tissue‐level stress or damage. Advanced models, such as musculoskeletal modeling or finite element analyses, would be required for this. Further, joint mechanical work and waveform‐level analyses may offer complementary insight into intra‐cycle joint mechanics, particularly in performance contexts. However, the present study focused on joint moments and fatigue‐weighted cumulative loading as metrics more directly related to joint loading magnitude and exposure relevant to KOA. Finally, the study focused solely on cycling and did not include longitudinal outcomes or direct comparisons with other locomotor tasks, limiting conclusions about long‐term effects or the relative loading of daily activities. The extent to which weighted cumulative moment measures relate to structural OA progression or symptom change remains unknown. Future work should therefore contextualize the cumulative loading metrics in longitudinal studies and by comparing KOA patients and controls across different movement types. Such comparisons would provide critical insight into whether cycling imposes higher, lower, or comparable cumulative loads and thereby strengthen the translational relevance of cycling as a therapeutic exercise.

## 5 Conclusion

This study demonstrates that instantaneous knee joint loading during cycling is best explained by crank torque, while cumulative loading is primarily determined by power output. Cadence plays a more specific role in cumulative knee extension loading, with higher cadences at a given power output increasing cumulative knee extension moments. These findings indicate that peak‐based instantaneous measurements alone do not fully characterize knee joint loading during cycling and that cumulative load metrics provide additional, clinically relevant insight into longitudinal joint exposure.

Importantly, this cumulative perspective also reveals group‐specific differences: individuals with KOA exhibit altered knee joint loading patterns compared with controls, mainly characterized by greater sensitivity of frontal‐plane loading to mechanical demand. From a practical perspective, minimizing cumulative knee joint loading, both in healthy cyclists (independent of age) and, more critically, in individuals with KOA, should focus primarily on controlling overall power output. Cadence may be adjusted according to comfort or performance needs, but sustained high cadences may increase cumulative knee extension loading and should therefore be considered carefully.

Overall, incorporating both cumulative load metrics and group‐specific loading patterns provides a more comprehensive basis for designing cycling‐based exercise strategies aimed at supporting knee joint health in individuals with and without knee osteoarthritis.

## 6 Perspective

Future research should address the largely unknown relationship between wCKL and disease progression in KOA. A critical prerequisite for this is the direct comparison of wCKL across different aerobic exercises, such as cycling, walking, and running. Such cross‐task comparisons provide a mechanical basis for positioning cycling relative to weight‐bearing activities and for informing activity selection and dosing in clinical practice. Building on this foundation, establishing whether cumulative load exposure predicts or correlates with structural and symptomatic changes in individuals with KOA could substantially improve the understanding of load patterns and guide exercise prescription. Further methodological advances that combine cumulative load metrics with subject‐specific modeling and clinical outcomes, as well as the inclusion of broader populations, will be essential to translate biomechanical insights into clinically actionable strategies.

## Funding

This project was funded by the Internal Research Funds of the German Sport University Cologne, grant agreement number L‐11‐10 011‐283‐093000. The funding source had no involvement in the study design, data collection, analysis, or interpretation, writing of the report, the decision to submit the article for publication, or any other aspect of the research.

## Conflicts of Interest

The authors declare no conflicts of interest.

## Data Availability

The data and analysis scripts that support the findings of this study are publicly available in the Open Science Framework (OSF) at https://doi.org/10.17605/OSF.IO/DKP4J.
